# An interview with Philip McTernan, section editor for the basic science section

**DOI:** 10.1186/2052-9538-1-3

**Published:** 2014-02-19

**Authors:** Philip G McTernan

**Affiliations:** University of Warwick, Warwick Medical School University Hospitals Coventry and Warwickshire, Clifford Bridge Road, Coventry, CV2 2DX UK

## Abstract

Philip McTernan is a Reader Associate Professor within Warwick Medical School at the University of Warwick. His main interests involve understanding the causes and underlying mechanisms of obesity mediated type 2 diabetes and investigating potential therapeutic targets. He is currently researching the origins of inflammation in human adipocytes and is the Section Editor for the new ‘Basic Science’ section in *BMC Obesity*. In this interview we find out a little more about the key issues in this area of obesity research.

## How did you first become interested in obesity research?

My earliest interaction with obesity-related research was in university during my first undergraduate biochemistry year, in the first ever lecture I attended at university. During this lecture I was subjected to an overview of the myriad of metabolic pathways I would need to learn and the challenges ahead to secure my degree. I almost ran from the lecture theatre there and then. Particularly as we were informed we would have to learn all of these human metabolism pathways in significant detail. This was back in the late 1980’s before molecular biology had taken hold in my world.

Looking back at this key personal event for me in the lecture theatre, I considered the scale of the learning task and all those eminent scientists that had brought to coalesce this knowledge in front of me. I literally did think, do I run for the hills or take it on? I chose the latter as you may have suspected, partly as the exit out of my row was blocked either way, partly because my biochemistry lecturer was a formidable character, and lastly because maybe I could do it. Little did I realize then how relatively simple the knowledge I was required to absorb, learn and regurgitate would be as the world of obesity was set to explode with research in so many new areas. However, during my undergraduate degree adipose tissue was then viewed as a storage tissue and an apparently dull one, and so attracted comparatively little interest from my then lecturers, in terms of comment or discussion. In fact, the world of obesity only came into my line of fire during my first postdoctoral research post. I remember being asked in the interview a lot of technical questions on my molecular and cell culture knowledge, whilst my questions to the panel were more based around the actual project on human adipose tissue. I soon realised why the panel were keen on my technical skill; no one had much expertise yet in culturing isolated human adipocytes as they are tricky floating cells to keep happy. I relished the prospect of the role however, as it gave me time to gain extensive knowledge on working with human adipose tissue and isolated adipocytes, and to study the effects of perturbing these cells in different ways with a clinical lean.

## Could you tell us about the career path that led you to your current position and research interests?

From my first postdoctoral research fellow post in the mid 90’s I joined the field of obesity research, following my doctorate on leukaemic therapies. I had appeared to have left the mainstream cancer field to join the back waters of adipose tissue metabolism. I even remember a conference in Dublin, where Professor O’Rahilly gave a lecture to what was then a relatively small crowd of die-hard obesity researchers, in which he discussed his apparent many long years in the back waters of obesity research, with the concluding remarks that “the times were a-changing”. Little did I realize then how right he was and how fortunate I was to be working in the obesity field with some under-valued yet bright scientists from across the world. Perhaps due to my lack of knowledge or experience but life as a young postdoctoral research fellow in the field of obesity began to feel a bit like working in the ‘Wild West’. To me it appeared like a new frontier with endless possibilities for both what could be done and how we could do it, as molecular biology techniques began to provide quick new information to somewhat overshadow the prior arduous and time-consuming studies.

In the field of obesity the story of leptin appeared to be a Pandora’s box of new possibilities. As a team we began exploring what these human adipocytes could produce, what would regulate adipokine production as well as proliferation, and how therapeutics might influence their metabolic functions. It was an exciting time as fat cells seemed to be incredibly versatile cells without the burden of decades of prior history as no one knew what they did. Our research began examining whether the adipokines noted in mice were expressed or relevant in human adipose tissue, and how conditions such as obesity and diabetes affected their regulation. Studies by the team examined how insulin influenced, and agents such as thiazolidinediones impacted adipokine function. The research became more aligned to the clinical end with a particular focus on this angle, prior to the economic and funding pressures to do translational research becoming apparent.

In terms of my current research, I continue to pursue my interest into the role of inflammation and the impact that metabolic state has on adipocyte cell function. I also have a continuing interest in nutrition, especially micronutrients and new health interventions concerning these, working to understand their impact on reducing inflammation and insulin resistance. Like most researchers, I am also always interested in new developments in the field of obesity and their implications on our current studies.

## What changes have you seen over the years in how obesity research is conducted?

Science’s capability and our understanding of obesity - its impact on health as well as our molecular understanding of cellular mechanisms, seems to have increased at an unprecedented exponential pace.

It was during the 90’s that these apparently ‘backwater’ obesity scientists were making heady new insights that would change the landscape of obesity research forever. In those early days the research was not always readily translatable, yet key to our fundamental understanding was the potential impact adipose tissue could have on health. We live in a different world today, as obesity is a readily identifiable global health problem for anyone to see. Human obesity is posing significant financial challenges to governments and health care providers, since the majority of us, myself included, are getting fatter day by day. To me it seems even more imperative that we do align the research to be translatable to human health. Yet it is also clear that we also do still need research that may not be readily translatable. We need new research ideas in this field that are away from the norm, with concepts that challenge our perspective and mainstream thoughts as you never know when a bit of ‘backwater’ research may become the new hot topic and change the world.

Funding bodies in recent years have often been keen to see the readily translated consequences from any obesity research undertaken. Even from a fundamental science perspective, basic science grant authorities always seem to require a section or an opening paragraph that has to align back to health consequences – but without stepping over the line; often a tricky business to manage. This change has not only occurred due to economic pressures resulting in reduced government and charitable basic science funding, but also due to the global explosion in obesity and its myriad of metabolic health consequences, all of which need studying. The demands of modern research appear different to the mid 90’s when I began in this field; today we spend increasingly less time at the bench and more time analysing the data (although this could be said of every generation). We certainly produce data much faster than ever before and with a much improved recognition of other specialities (ranging from mathematical modelling, Systems Biology, Engineering and digital healthcare), leading to a more interdisciplinary approach to science. However, for the die-hard bench worker who should never be forgotten, it is true to say that whilst many technological advances have occurred, cell culture and animal studies can still remain relatively slow and laborious work.

## What do you feel are the main advances that have been made in our research over the recent years in our quest to understand the causes and links of metabolism and genetics with obesity – which advances have particularly interested you?

I should say that genetics has never been a keen strength of mine. As an undergraduate student my co-worker and I were tasked to undertake the classic drosophila genetics experiment to cross white-eyed flies to wild-type red eyed flies to create an F1 hybrid and so on. An excellent genetics lesson as it provides a good example with which you can assess the impact of a trait that depends on a single gene. With the parental cross all were red eyed, and in the F1 back cross with white eyed parental-type there were due to be four colours (red eyes, bright red eyes, brown and white eyes). However, at some stage between the parental cross, and F1 back cross, I remember we managed to produce a one eyed white mutant drosophila that was not listed at all. My lecturer was not impressed. I considered at that point that an inability to master drosophila would present significant challenges if I ever considered embarking on trying to understand the complexity of human genetics. However, despite this I feel that monogenic human obesity has shown us the extreme phenotypes (which are rare when compared to the more common form of obesity), and these offer a unique insight into obesity as a whole. Studies in mice show that these phenotypes highlight that even in these cases humans are not a simple species.

Polygenic obesity by its nature has been a difficult case to crack in terms of locating those genes that appear to raise their head above the significance line. Polygenic obesity association studies assessing single nucleotide polymorphisms (SNPs) have noted that many lie within the first intron of the fat mass– and obesity-associated gene (FTO) which is strongly associated with adiposity. In the initial stages before we knew that FTO encoded for a 2-oxoglutarate (2-OG) Fe^2+^-dependent dioxygenase, FTO showed us a revitalising glimpse of how relevant genetic studies are - despite the large scale heterogenic complexity of humans. However, it’s clear that understanding the functionality aligned with these genes, challenging though it may be, is vital for continuing studies. Whilst we know that genes affect our body fat we also now realize that the naive view that adipose tissue is just a simple storage organ has certainly gone.

The molecular biology biomarker era and the more recent epigenetic studies that have been conducted have certainly given us food for thought on our way to discovering individual risk profiles. Yet significant new research programmes have decided we should also consider more closely what food we eat, and look at moving towards eating more ‘superfoods’, *i.e*. using functional food value as a potential diet related treatment option to reduce the obesity mediated metabolic risk for ourselves and offspring. It must seem like a return to previous times, to understand the benefits that food in an unprocessed manner may have had on us and our children, having realised that previous generations were healthier than current and future ones will be. This doesn’t mean to say this avenue will be easy, as diets, gut flora and cellular metabolism thrown together may present quite a challenge to embark on even in this time of modern technologies.

## What are the new areas of research that are happening now which you feel are particularly important?

Focusing on gut flora, I am informed reliably that everyone has around 1000 bacterial species lining our intestines at any one time, with 60 % of our dry stool mass being made up of microbes. Adding to this the fact that at any one time the people around you at work may have very different bacterial species diversity or gut flora signatures, makes you wonder how varied all our gut floras may be. Now for example throw people onto a plane in a confined space for several hours from different countries, different homes, and different diets, then you could have up to 10,000 different species being passed around changing the species diversity and bacterial species proportion in your gut. Not of course forgetting the prior passengers and the bacteria they may have left behind. Also spare a thought for the flight crew who may be receiving and passing around a lot more than just the drinks.

But why does this matter for obesity research? Well, although data-wise this research is in its relative infancy, what our gut flora constitutes of or what our metabolic gut flora signatures look like, may have quite a profound effect on our health - both in terms of the capabilities of the gut flora’s action within the gut, and the impact of gut derived gram negative bacterial cell wall fragments, also known as endotoxin, that can pass across the gut mucosa inducing undesired inflammatory responses and tissue damage. Furthermore, your gut flora could be influenced by as little as a single meal which may end up impacting your health longer term than a more recent meal. Both the effects of the gut flora in the intestine and systemically may have implications for metabolic dysfunction and disease risk. Currently the NIH has 16 out of its 27 centres with programmes funding gut microbiome work in some form or other and the impact this has on our health. This is clearly a hot topic area for the present and future few years and if there was ever a time to need big data capability, analyzing the human gut flora surely must be it, as it will be a huge and complicated task not for the faint hearted or those easily unsettled by turbulence on the route ahead.

## Why do we need a new open access, broad scope obesity journal – what will *BMC obesity* be bringing to the obesity research community?

It is clear that there are a number of high profile diseases that governments and funders are focussing on, and rightly so. Interestingly, obesity is often in the background as a contributing cause to what at times seems like almost every disease. This being the case, and coupled with the fact that obesity has a complicated environmental heterogeneity, means that it remains a very difficult disease to tackle. Additionally, there is also the creeping realisation that our obesity doesn’t just affect our own health but that of our children in the next and subsequent generations. Even if our future relatives won’t know us except through a family tree, they may get more from us than they bargained for. All of this means that the obesity field has multiple challenges - understanding the impact of nutrition on our systemic health, its epigenetic impact, as well as identifying key genetic traits that may change with our environment and help to further obesity mediated metabolic disease. As well as not forgetting the diversity of the enduring adipocyte and the new realisation that adults appear to have brown fat.

Brown fat is important as the brown adipocyte cell is unlike the white in that it contains an abundance of mitochondria which dissipate energy readily by generating heat for the organism through non-shivering thermogenesis. It’s clear that nutrition, gut flora, inflammation and epigenetics are big news for now and the future. On this basis it is important to have a journal in the *BMC* series that can focus exclusively on the rapidly growing field of obesity, highlighting novel findings through the online open access platform of the series and under a journal name that people trust.

## Where do you see the obesity research field heading – what does the future hold and what work is still to be done?

A common question I am asked when speaking to the public or keen students on obesity research, or even friends outside of work, is how far along the road in obesity research we are. In reply I initially draw upon an old joke, sometimes sent to friends in their birthday card. The card depicts a person sitting on the side of the hill looking over the landscape. The caption underneath reads, ‘you’re not over the hill yet but you’ve good a pretty good view’. I would use this analogy to depict how far we have come in obesity research and how far we have to go - although some may argue we already know so much.

Whichever such ideas that we have had already, patting ourselves on the back for considering how clever we may have been, reminds me of the story of a US patent office official who resigned and recommended that the patent office be closed back in the 1840's as he thought that everything that could possibly be invented had already been done! Whilst this has been a funny story passed around the globe, it appears to be just a myth. There was no apparent patent officer who resigned and in fact this story may have been taken out of context from a report from the Patent Office Commissioner Henry Ellsworth 1843 report to US Congress, who suggested that “The advancement of the arts, from year to year seems to presage the arrival of that period when human improvement must end.” However, the original myth seems to perpetuate much more than the commissioner’s comments. Each generation may now consider that they have been the brightest and cleverest people to consider such novel new insights.

This analogy could be easily used in the field of obesity. Haven’t we discovered the main pieces of evidence to solve the problem? It is true that we have made significant advances and to a certain extent one could argue we know what we should do to reverse obesity through surgical and weight loss intervention programmes. However, we still aren’t good at working out individual personal risk profiles to manage the individual effectively or providing a long-term cost effective treatment platform considering the obesity pandemic.

Certainly it seems that future obesity research will invest heavily into determining the impact of functional foods on our health, understanding the value of food produce - their extracts and even individual components. Particularly as food manufacturers are even keener to investigate what health benefits their foods may have on us all. So does obesity research seem to align to that mythical patent office official who said it was time to pack up and go as there are no new advances? Absolutely not, as whilst it is true there has been an upscale in functional food research we now have much more sophisticated tools to be able to understand what is going on and to address whole body metabolism as well as at the molecular level. Furthermore, if you are one of those researchers who haven’t noticed the many new nutritional and health departments or institutes and staff title changes that have sprung up in this area, then where have you been?

Obesity research which has led to discoveries of the links between specific genes such as FTO and obesity, microRNAs and the change in our understanding of adipose tissue, amongst others, has not only been highly interesting but at times shocking. Therefore, as we look out on the side of the hill considering how far we have travelled in understanding obesity, are we further up or lower down the hill? Very few may agree exactly where we are and how long it might take us to reach the peak or final destination. In terms of treatment and weight loss interventions, will we pursue weight loss drugs, as well as ways of reducing metabolic risk without weight loss via a functional food or other diet interventions? Will we use prevention programmes with obesity never becoming apparent? We know that for those patients with morbid obesity bariatric surgery is often a way to limit multiple co-morbidities - although this is not feasible for all. For most of the population where diet is the key it is apparent we need ways to necessarily fool our bodies into thinking or feeling full, whilst considering we may not be dieting! Part of the solution in our diet is also trying to ensure that what we eat doesn’t aggregate our tissues into a pro-inflammatory state, so that perhaps we can live with a bit more fat without the metabolic complications. This may arise with diets including functional foods which may limit systemic inflammation.

The question of what the future holds depends largely on which obesity strategies we will ultimately deliver to patients as this changes where we might consider ourselves to be sitting on the side of that hill and the view from it, which currently still appears up for grabs. And in response to speaking to the public, students and friends, I never say how high up the hill we are or how long it might take to get to the peak, just that the outlook is good!
